# Multidimensional Clinical Assessment in Frontotemporal Dementia and Its Spectrum in Latin America and the Caribbean: A Narrative Review and a Glance at Future Challenges

**DOI:** 10.3389/fneur.2021.768591

**Published:** 2022-02-16

**Authors:** Fernando Henríquez, Victoria Cabello, Sandra Baez, Leonardo Cruz de Souza, Patricia Lillo, David Martínez-Pernía, Loreto Olavarría, Teresa Torralva, Andrea Slachevsky

**Affiliations:** ^1^Geroscience Center for Brain Health and Metabolism (GERO), Santiago, Chile; ^2^Memory and Neuropsychiatric Clinic (CMYN) Neurology Department, Hospital del Salvador and Faculty of Medicine, University of Chile, Santiago, Chile; ^3^Neuropsychology and Clinical Neuroscience Laboratory (LANNEC), Physiopathology Department – Institute of Biomedical Sciences (ICBM), Neuroscience and East Neuroscience Departments, Faculty of Medicine, University of Chile, Santiago, Chile; ^4^Laboratory for Cognitive and Evolutionary Neuroscience (LaNCE), Department of Psychiatry, Faculty of Medicine, Pontificia Universidad Católica de Chile, Santiago, Chile; ^5^Universidad de los Andes, Departamento de Psicología, Bogotá, Colombia; ^6^Programa de Pós-Graduação em Neurociências da Universidade Federal de Minas Gerais (UFMG), Belo Horizonte, Brazil; ^7^Departamento de Clínica Médica, Faculdade de Medicina da Universidade Federal de Minas Gerais (UFMG), Belo Horizonte, Brazil; ^8^Departamento de Neurología Sur, Facultad de Medicina, Universidad de Chile, Santiago, Chile; ^9^Unidad de Neurología, Hospital San José, Santiago, Chile; ^10^Center for Social and Cognitive Neuroscience (CSCN), School of Psychology, Universidad Adolfo Ibáñez, Santiago, Chile; ^11^Institute of Cognitive and Translational Neuroscience (INCYT), Instituto de Neurología Cognitiva Foundation, Favaloro University, Buenos Aires, Argentina; ^12^Department of Neurology and Psychiatry, Clínica Alemana-Universidad del Desarrollo, Santiago, Chile

**Keywords:** frontotemporal dementia, neuropsychological assessment, functional assessment, gait assessment, behavior assessment, neuropsychiatric symptoms, multidimensional assessment, consortium

## Abstract

Frontotemporal dementia (FTD) is the third most common form of dementia across all age groups and is a leading cause of early-onset dementia. The Frontotemporal dementia (FTD) includes a spectrum of diseases that are classified according to their clinical presentation and patterns of neurodegeneration. There are two main types of FTD: behavioral FTD variant (bvFTD), characterized by a deterioration in social function, behavior, and personality; and primary progressive aphasias (PPA), characterized by a deficit in language skills. There are other types of FTD-related disorders that present motor impairment and/or parkinsonism, including FTD with motor neuron disease (FTD-MND), progressive supranuclear palsy (PSP), and corticobasal syndrome (CBS). The FTD and its associated disorders present great clinical heterogeneity. The diagnosis of FTD is based on the identification through clinical assessments of a specific clinical phenotype of impairments in different domains, complemented by an evaluation through instruments, i.e., tests and questionnaires, validated for the population under study, thus, achieving timely detection and treatment. While the prevalence of dementia in Latin America and the Caribbean (LAC) is increasing rapidly, there is still a lack of standardized instruments and consensus for FTD diagnosis. In this context, it is important to review the published tests and questionnaires adapted and/or validated in LAC for the assessment of cognition, behavior, functionality, and gait in FTD and its spectrum. Therefore, our paper has three main goals. First, to present a narrative review of the main tests and questionnaires published in LAC for the assessment of FTD and its spectrum in six dimensions: (i) Cognitive screening; (ii) Neuropsychological assessment divided by cognitive domain; (iii) Gait assessment; (iv) Behavioral and neuropsychiatric symptoms; (v) Functional assessment; and (vi) Global Rating Scale. Second, to propose a multidimensional clinical assessment of FTD in LAC identifying the main gaps. Lastly, it is proposed to create a LAC consortium that will discuss strategies to address the current challenges in the field.

## Introduction

Frontotemporal dementia (FTD) is a clinical neurodegenerative syndrome characterized by alterations in behavior, executive functions, and language (1–3). The FTD constitutes a spectrum of diseases classified according to their clinical presentation and patterns of neurodegeneration (4, 5). There are two main types of FTD: the first is the behavioral FTD variant (bvFTD), characterized by impaired social function, behavior, and personality; and the second are the language variants, namely, semantic dementia (SD), non-fluent or agrammatical aphasia (nfv-PPA), and logopenic aphasia (lv-PPA), which are characterized by progressive deficits in language skills (2, 4, 6). There is a current controversy surrounding lv-PPA, regarding whether to maintain its inclusion as an FTD variant, given that the neuropathological studies show a stronger association with Alzheimer's Disease (AD) pathologies (7, 8). Nevertheless, some current criteria maintain it as an FTD syndrome variant (6). Other types of FTD-related disorders present with motor symptoms and/or parkinsonism. The main disorders associated with motor difficulties are FTD with motor neuron disease (FTD-MND) and FTD with atypical parkinsonism, i.e., progressive supranuclear palsy (PSP) and corticobasal syndrome (CBS) (2, 9, 10).

FTD is one of the most common causes of early-onset dementia (patient age <65 years) and is the third leading cause of dementia after AD (11, 12) and Lewy body dementia (LBD) (1). Its prevalence ranges between 3 and 26% worldwide (1, 13). Precise data regarding its prevalence in Latin America is unknown despite the consequences it causes (14). It is also frequently underdiagnosed, being confused with psychiatric pathologies (15, 16). Studying this syndrome is greatly relevant as it impairs the capacity of the patient to perform activities of daily life (ADL), affecting both basic (feeding, dressing, and bathing) and instrumental (economic management, cooking, housework) activities of daily living (BADLs and IADLs, respectively) (17, 18). This significantly interferes with the capacity of the patient to live independently, their quality of life, along with that of their relatives (19, 20).

Diagnosis is based on identifying the clinical phenotype described above, i.e., behavioral or neuropsychiatric symptoms and/or language impairment, accompanied by impairment in other domains, namely, social cognition, executive functions, functionality, and motor function (2). The clinical interview and examination are complemented by a multidimensional assessment, defined as the evaluation of cognition, behavior, functionality, and motor capacity, with the administration of validated and standardized tests and questionnaires to obtain reliable and accurate information regarding impairment in these domains (21). Broadly speaking, these tests and questionnaires could be administered in the clinical context as a brief screening evaluation, but they can also be a complementary exam when applied as an extensive neuropsychological assessment (22). Cognitive screening tests are brief and straightforward instruments aimed at detecting signs of dementia or cognitive impairment and monitoring the evolution of the disease and response to treatment (22, 23). These instruments are routinely used in a clinical practice. They are crucial for identifying cognitive impairment and for initiating the diagnostic process, which is further supported by blood tests, neuroimaging, and a formal neuropsychological assessment (22, 23), which includes an evaluation to collect information on various dimensions of cognition, behavior, and functioning (24). The validity and reliability of data gathered with brief screening tests and neuropsychological tests depend on their validity in the cultural contexts in which they were applied (25, 26). A test with good psychometric characteristics allows comparing the performance of a subject with groups of the same age, sex, race, and educational level, given that all these factors influence the performance and interpretation of the instruments used. This comparison determines whether a subject performs as expected or with diminished capabilities, which can be quantified and interpreted (24). Although the screening tests are a powerful tool to detect cognitive impairment, there is no specific screening for FTD due to the heterogeneity of the syndrome, which implies a significant difficulty for a timely diagnosis.

Diagnosing FTD is indeed challenging due to its complex clinical phenotype and its insidious presentation, especially in cases with non-specific behavioral features and without brain atrophy (27–31). Usually, an FTD diagnosis is clinically recognized later than AD (15, 16, 32). A significant delay in diagnosis of up to 5 years from the onset of the first symptoms and a high rate of misdiagnosis with psychiatric conditions have been reported (33, 34). Several diagnotic barriers have been reported, such as (i) the heterogeneity of FTD, whose clinical features frequently overlap with other neurological diseases, e.g., the behavioral/dysexecutive variant of AD (35) or psychiatric disorders (36–39); (ii) Lack of knowledge and training of health professionals in Latin America and Caribbean (LAC) on FTD (25, 40, 41); (iii) Limited access to medical care, neuropsychological evaluations, and advanced neuroimaging facilities to support FTD diagnosis in LAC (42, 43); and (iv) Lack of validated instruments for the LAC population that is capable of detecting and differentiating FTD from other pathologies. For these reasons, it is important to review the available evidence on tests and questionnaires for the assessment of FTD in LAC and propose a strategy to address challenges in the field.

Therefore, our paper has three main goals. First, to present a narrative review of the main tests and questionnaires published in LAC to assess FTD and its spectrum. Second, to propose a multidimensional clinical assessment of FTD in LAC, identifying the main gaps. Lastly, it is proposed to create an LAC consortium that will discuss strategies to address current challenges in the field.

## Methods

First, experts in FTD and its spectrum from Argentina, Brazil, Chile, and Colombia were invited to participate based on two criteria: (i) neurologists, neuropsychologists, and physical therapist working in clinical evaluation and research in FTD and its spectrum, or (ii) clinical researchers in the clinical assessment of FTD and its spectrum. Second, an online literature search for journals indexed by Pubmed Central, Scopus, Lilacs, and Scielo databases was conducted between March 2021 and July 2021 (performed by FH and VC). The Scielo database was incorporated since it indexes many national and Latin American journals from all areas of knowledge. For this review, we searched for articles with the following keywords in English: Frontotemporal Dementia, Primary Progressive Aphasia, Progressive Supranuclear Palsy, Corticobasal Degeneration, Amyotrophic Lateral Sclerosis AND Neuropsychology, Neuropsychiatric, Activities of Daily Living, Functional Assessment, Cognitive Assessment, Screening Test, Gait, Behavior, AND Latin America, South America, Caribbean. Subsequently, the procedure was reproduced with the exact keywords translated into Spanish and Portuguese.

Once the results of the literature review were provided to the experts, they wrote the different sections of the narrative review based on their expertise (LCD, LO, AS, and FH: cognitive screening; SB and TT: neuropsychology assessment; DMP: gait assessment; PL and FH: behavior and neuropsychiatric symptoms; and FH and AS: functional assessment and global rating scale). After the experts wrote the different sections, they met in several online meetings to reach an agreement on the different sections of the narrative review, and to propose a multidimensional clinical assessment and identify the main gaps in the field.

## Results

### Description of Available Evidence for Multidimensional Assessment in LAC

In the following section, we will present the available evidence divided into six dimensions: (i) Cognitive screening; (ii) Neuropsychological assessment divided by cognitive domain; (iii) Gait assessment; (iv) Behavioral and neuropsychiatric symptoms; (v) Functional assessment; and (vi) Global rating scale. We will discuss the relevance of each dimension for the assessment in the FTD diagnosis, describing the instruments generally used along with the available evidence in LAC.

#### Brief Cognitive Screening

As discussed previously, FTD diagnosis is based on clinical grounds and requires a high level of suspicion from health professionals. When evaluating a patient with suspected dementia, a brief cognitive screening (BCS), defined as an instrument used to detect signs of dementia that does not include caregiver or information interviews, is the first line of cognitive assessment (23). BCSs are crucial for identifying the presence of a cognitive syndrome, initiating the diagnostic process, and contributing to a timely diagnosis (44).

However, there are no specific tools for screening for neurodegenerative syndromes. In line with this, epidemiological surveys on the prevalence of FTD in community-based studies in LAC employed a three-step procedure to establish FTD diagnosis, namely, (1) demographic and clinical questionnaires, including a brief cognitive battery, e.g., Mini-Mental State Examination (MMSE) (45), Montreal Cognitive Assessment (MoCA) (46), third version of Addenbrooke's Cognitive Examination (ACE-III) (47), and a functional assessment such as the Pfeffer Functional Activities Questionnaire (PFAQ) (48); (2) detailed clinical (neurological) and cognitive evaluations, and (3) laboratory and neuroimaging investigation. Thus, FTD diagnosis is established with a consensus diagnosis (14). Moreover, most clinical studies on FTD conducted in LAC included patients selected from the reference centers to diagnose and manage dementia. These studies usually adopt a consensual diagnostic framework. Indeed, cognitive screening tests are recommended for detecting dementia but not for the differential diagnosis of dementia. Thus, it is crucial to use cognitive screening tools sensitive to FTD (49).

BCS is generally used in FTD research, such as the INECO Frontal Screening (IFS) (50), the Frontal Assessment Battery (FAB) (49, 51), or the Mini-social cognition and emotional assessment (mini-SEA) (52, 53). In addition, the behavioral and psychiatric scales answered by an informant, such as the Neuropsychiatric Inventory (54), may also be helpful for FTD diagnosis (55, 56). However, these tools may not be adapted for use in primary care scenarios as they may require specialized training and are time-consuming (23). Moreover, their accuracy for FTD screening in the general population has, so far, not been investigated.

This context, thus, warrants the development or adaptation and validation of screening tools for FTD diagnosis. The ideal FTD screening tool should combine high sensitivity and short application time and should not require specialized training, thus, being beneficial for primary care settings.

In LAC, brief cognitive assessments are available for use in clinical settings. However, evidence on their diagnostic utility in FTD is still limited. Addenbrooke's Cognitive Examination-Revised (ACE-R) was adapted in Argentina (57), Brazil (58), and Chile (59). Another work conducted in Argentina and Chile has validated the third version of Addenbrooke's Cognitive Examination (ACE-III) in a population of patients with bvFTD, AD, and healthy control subjects (60). The ACE-III showed good psychometric properties and allowed differentiating patients with dementia from healthy controls, and demonstrated good discriminative ability between these two groups of patients (60).

Torralva et al. (50) designed the IFS in Argentina, a cognitive instrument that allows a brief assessment of executive functions. The validity and discriminative capacity of the IFS was studied in patients with bvFTD, AD, and healthy controls. The IFS differentiates patients with dementia from healthy controls (50, 61) and patients with bvFTD from AD (50). Two studies, one in Argentina and the other in Peru, suggested that the IFS presented greater clinical utility in differentiating bvFTD from AD in comparison with the FAB (62, 63). In Brazil, Bahia et al. (64) reported that the IFS showed good psychometric properties, but provided a low accuracy, differentiating between bvFTD and AD. In Chile, the psychometric properties and diagnostic accuracy of IFS were studied in a sample of patients with dementia (bvFTD, AD, vascular dementia (VD), LBD, and SD) and healthy controls (65). The Chilean IFS presented adequate indicators of reliability and good diagnostic accuracy in detecting patients with dementia (65).

#### Neuropsychological Assessment Divided by Domain

##### Memory

Although relative sparing of episodic memory has been proposed as one of the distinctive characteristics of FTD (66, 67), recent evidence questions the validity of the preservation of this domain, particularly in bvFTD. For instance, evidence from a recent meta-analysis (68) showed that patients with bvFTD perform intermediately between healthy controls and patients with AD. However, patients with bvFTD showed severe memory impairments in line with previous studies reporting episodic memory impairments in patients with bvFTD (69, 70). In contrast, several studies demonstrate that patients with AD experience even more significant memory problems than patients with bvFTD (71–74), with delayed memory testing being the most discriminative (73, 75). In addition, some patients with bvFTD have shown genuine amnesia affecting storage and consolidation abilities, which are independent of executive dysfunctions (76), and are observed in a similar degree in AD (77, 78).

Concerning PPA, episodic memory seems to be compromised in all variants compared to healthy controls (78, 79). However, patients with SD are impaired to a similar extent as patients with lv-PPA who are in turn more impaired than patients with nfv-PPA. In addition, patients with SD perform better on tests using non-verbal material and show overall better performance on recognition tests (78). Episodic memory deficits in lv-PPA and nfv-PPA, on the other hand, are observed on both verbal and non-verbal measurements, although patients with lv-PPA show more pronounced episodic and working memory deficits when compared to patients with nfv-PPA (79–81). Thus, given that differentiating the language profiles of the PPA variants remains challenging (80), especially for lv-PPA and nfv-PPA, memory testing could be of potential benefit to better differentiate between these variants.

The most frequent tests used to assess memory in FTD (82) are the Rey Auditory-Verbal Learning Test (RAVLT) (83) or similar word list-learning tests, such as the Hopkins Verbal Learning Test (HVLT-R) (84) or the California Verbal Learning Tests (CVLT) (85), the computerized Paired Associate Learning Test (PAL) (86), the Free and Cued Selective Reminding Test (FCSRT) (87), the autobiographical memory interview (88), and the Cambridge Behavioral Prospective Memory Test (CAMPROMPT) (89). These instruments are also commonly used in LAC [e.g., (52, 90)], although most of them are not validated for this population.

LAC validations are available for the RAVLT (91, 92), the HVLT-R (93), and the FCSRT (94). Other validated memory tests for the assessment of patients with dementia include the Rivermead Behavioral Memory Test (RBMT) (95) and the Logical Memory Subtest of the Wechsler Memory Scale (WMS) (96) for the Brazilian population, and the Signoret battery for amnesic efficiency (BEM 144) for the Argentinian population (97). In addition, the Short-term Memory Binding (STMB) test has been used to assess patients with bvFTD in Brazil (98). Results showed that patients with AD performed significantly worse than controls and patients with bvFTD in the STMB test, while both clinical groups showed equivalent performance. Therefore, this test can be used for clinical purposes and may aid in the differential diagnosis of AD (98). Finally, the visual memory test from the Brief Cognitive Screening Battery (99) has also been employed to investigate episodic memory of patients with bvFTD in Brazil (100).

In conclusion, findings suggest that clinicians should carefully use memory performances and interpret them in conjunction with other diagnostic information, namely, medical history, behavioral observations and questionnaires, neuroimaging, and neuropsychological data from other cognitive domains (68, 101).

##### Visuospatial and Constructional Skills

Visuospatial function is usually conceptualized in three components: visual perception, construction, and visual memory (101). The relative preservation of visuospatial abilities is suggested to be among the critical features that distinguish FTD from other degenerative disorders and, particularly, from AD (67) and LBD (102). However, a recent study (103) showed that the visuospatial measures demonstrate a limited ability to distinguish between AD and bvFTD unless disease severity is considered. Controlling for disease severity reveals a disproportionate visuospatial impairment in AD compared to bvFTD.

One of the most commonly used instruments to assess visual perception is the Visual Object and Space Perception Battery (VOSP) (104). In this battery, patients with the three language FTD variants obtain lower scores than controls, while patients with bvFTD perform normally (105). However, scores deteriorate with the dementia progression in all patient groups (105). Drawing tasks, such as the Clock Drawing Test (CDT) (106) and the Rey-Osterrieth Complex Figure (ROCF) (107) test, are commonly used to assess constructional abilities. Grossi et al. (108) found that patients with bvFTD and patients with AD achieve similar scores on copying tasks, present similar drawing procedures in the ROCF, and make a similar quantitative and qualitative pattern of errors when copying simple geometrical drawings, which suggests that relative preservation of visuospatial abilities in FTD may be found in early stages of the disease. Finally, some tests are widely used to assess visual memory, including the delayed recall component of the ROCF and the Benton Visual Retention Test (BVRT) (109). In this line, a comprehensive systematic review (110) found that ROCF recall and topographical memory tasks show the greatest diagnostic potential in dementia, while the BVRT shows potential as a prognostic marker.

Regarding the PPA variants, patients with lv-PPA have shown significantly lower scores on all visuospatial skills (111). The nfv-PPA variant shows significant difficulty in all visuospatial abilities except the delayed recall. In contrast, SD performs poorly only on delayed recall of visual information. The lower scores of all patients with lv-PPA on visuospatial skills could be explained by the fact that part of the clinical criteria for this disease includes parietal atrophy on structural MRI or hypometabolism on PET/SPECT (111). One possible reason patients with nfv-PPA displayed difficulty on these tasks is that several of the tasks rely on visuomotor abilities, and nfv-PPA has been associated with the degradation of white matter pathways connecting the left inferior frontal gyrus to the premotor and supplementary motor regions (112, 113). Thus, the deficits may relate more to motor planning and sequencing (111). Further, investigation is needed to determine the underlying mechanism.

Some of the most employed measures have been validated for LAC, including the CDT (114–118) and the ROCF test (119, 120). In addition, the VOSP has also been validated for the Brazilian population (121).

##### Language Assessment

Although language in bvFTD is initially spared (101), some patients with this variant may present difficulties in naming action words. Such a deficit has shown an association with executive abilities (122). In addition, due to apathy, patients with bvFTD may not participate in communication, and, thus, may present a reduction in spontaneous speech (101). Social and emotional aspects of speech may also be impaired in bvFTD, with an inability to understand the subtleties and context of conversations (123). Fluency may also be helpful in differentiating bvFTD and AD. While semantic fluency is usually impaired to a greater degree in AD, phonemic fluency is more affected in bvFTD (123).

Regarding PPA, the most prominent early feature of SD is a reduced expressive vocabulary. Word finding is severely impaired, and speech is empty of content (124). Compared to SD, the hallmark feature of nfv-PPA is effortful non-fluent speech. Nfv-PPA is characterized by grammatical errors and omissions, along with the simplification of grammatical forms (125). The third subtype of PPA, lv-PPA, is mainly characterized by problems in lexical retrieval during conversational speech and impaired repetition of sentences and phrases.

Tests of word comprehension, speech production (fluency, naming, and repetition), as well as oral reading (to detect surface dyslexia) and writing (to detect surface agraphia), should be used in the language assessment of FTD variants (82). The main instruments used for language assessment are the Boston Diagnostic Aphasia Examination (BDAE) (126) and the Sydney Language Battery (SYDBAT) (127). The SYDBAT contains four subtests: nomination, repetition, comprehension, and semantic association. The most commonly used instruments for the assessment of memory or semantic knowledge are the Pyramids and Palm Trees (PPT) Test (128), which measures the accessibility of semantic information of words and images, and the Repeat and Point Test (RPT) (129), which assesses the comprehension and repetition of words, differentiating patients with DS and nfv-PPA.

Some of these language measures have been validated for LAC. For example, normative data on the BDAE and verbal fluency tests exist for the LAC Spanish-Speaking Population (130) and for Brazilian Portuguese (131–135).

##### Praxis

Apraxia is one of the major sources of disability in patients with brain injury, as it significantly affects Activities of Daily Living (ADLs) (136). Although apraxia is a main sign of other neurodegenerative pathologies, such as CBS, it is also known to present as an additional early cognitive marker in bvFTD (137), and therefore, its assessment is important (138). Some findings also suggest a relationship between praxis and working memory in this type of patients, since frontal involvement, with its corresponding difficulties in executive memory, hinders the performance, for example, of gestures (137, 139). Additionally, there are FTD variants or diseases with overlapping symptoms where this function is particularly affected. For instance, PPA presents speech apraxia (140), and CBS is characterized by the presence of progressive and asymmetric apraxia (141–143).

Scientific evidence in LAC supports apraxia as an early manifestation of bvFTD and as the most significant manifestation in the previous variants described. Several of the findings on the subject have studied a positive relationship between the severity of apraxia and the degree of cognitive impairment (136).

The most commonly used tests to measure this function in FTD are the ROCF Test (83, 107), the CDT (144), the block design Wechsler Adult Intelligence Scale (WAIS) construction subtest (145), the Cognitive Assessment of Apraxias battery (146), and the Mattis Dementia Rating Scale (MDRS) (147). Some of these praxis measures have been validated for LAC. For example, normative data exist on the ROCF (119, 148), on the WAIS IV construction subtest with cubes (149), and on the MDRS (150–152). In addition, the Cognitive Assessment of Apraxias battery (153) was created in Argentina.

##### Executive Functions

Executive functions are defined as an umbrella concept, encompassing multiple functions commanded by the frontal lobe, such as planning, organization, sequencing, inhibitory control, and cognitive flexibility (154, 155). In FTD, their assessment is of vital importance as it implies the involvement of the prefrontal cortex and some of its variants present a dysexecutive profile (82, 156).

The most commonly used tests to measure this function in FTD can be of three types. Executive screening tests, such as the IFS (50, 157) discussed above, provides a general idea of the preservation or impairment of these functions. A group of classic executive functions assessment tests includes the Trail Making Test A and B (TMT) (158), the Wisconsin Card Sorting Test (WCST) (159), the Stroop test (160), the Hayling Test (161), the Tower of London (162, 163), the Tower of Hanoi (164), the Porteus Maze (165), Raven's Progressive Matrices Test (166), WAIS Matrix Reasoning subtests (145), Iowa Gambling Test (IGT) (167), and the classic working memory tests, such as the reverse digits, arithmetic, and WAIS letter ordering (145). Finally, there are ecological evaluation tests, such as the Hotel Test (168) and the Behavioral Assessment of the Dysexecutive Syndrome (BADS) (169), which optimally evaluate the functioning of the patient with tasks designed similarly to their daily life.

Some of these executive functions measures have been validated for LAC. For example, normative data exists on the TMT A and B (118, 170, 171), on the Modified Wisconsin Card Sorting Test (M-WCST) (172, 173), on the Stroop Color-Word Interference Test (173, 174), on the executive subtests WAIS IV (149), on the Hayling Test (175, 176), on the BADS (177), and on the Hotel Test (52, 178). In addition, the IFS was created in Argentina (50).

The existing scientific evidence in Latin America predominates in patients with bvFTD, who, in addition to behavioral symptoms, present a predominant dysexecutive profile in the neuropsychological assessment (179–181).

##### Social Cognition

Social cognition refers to the set of cognitive processes involved in the perception, interpretation, and generation of responses to the intentions, dispositions, and behaviors of others (182). This domain plays a very relevant role in FTD as it is predominantly affected in the behavioral variant, one of the most common variants of FTD, particularly regarding recognition of emotions, theory of mind, empathy, and moral judgment tasks. These failures occur mostly due to the effects on the orbitofrontal cortex and temporal poles (183–187). Various findings highlight difficulties, such as impaired moral judgment, where patients with FTD score are significantly lower on personal moral dilemma tasks and theory of mind tests than the control subjects (183). In addition, other studies suggest that patients with FTD judge intentional damage as more permissible than accidental damage due to a decrease in gray matter in the temporal pole (188). Investigations studying empathy in this group of patients are also especially relevant, finding that patients with FTD present difficulties in the affective, cognitive, and moral aspects of empathy (184).

Therefore, the most commonly used tests for evaluating these difficulties in social cognition are the Facial Expressions Recognition Test (189), the Mind in the Eyes (190), the Faux Pas Test (191), the Social cognition and Emotional Assessment (SEA) (192), and the short version of the Social Cognition and Emotional Assessment (Mini-SEA) (193). Some of these praxis measures have been validated in LAC, or new versions have been created, such as the Facial Expressions Recognition Test for elderly Argentinians (194) and the Facial Emotions Recognition Test in Brazil (195). In addition, normative data exists on the Mind in the Eyes (52, 196), and the Faux-pas tests (52). The Faux-pas test has also been adapted in Brazil (197) and used for bvFTD investigation (198).

Numerous studies on social cognition in patients with FTD have been carried out in LAC, especially the relation to moral judgment, theory of mind, and the recognition of emotions (53, 183–187, 198, 199).

#### Gait Assessment

Motor control has long been understood as a mechanical function and reflex, but an extensive body of research shows that motricity depends on different cognitive processes, such as attention, memory, language, and executive function (200, 201). Especially relevant in motor assessment is the study of gait. Gait is a complex task integrating the participation of multiple systems in order to achieve a cyclic pattern of body movements with cognitive function (202, 203), encompassing multiple independent domains [e.g., pace, rhythm, variability, asymmetry, and postural control (204)]. Gait analysis has shown to be a good predictor for health status in older adults and is a global health marker (205, 206). In the dementia population, studies have shown a strong association between gait and cognition (207) where an assessment according to serial quantitative measures of gait velocity prove to be a good predictor of dementia development (208).

Gait speed has been one of the most reported locomotion variables because of its robust properties in clinical settings (209) and its utility in differentiating between healthy older adults and patients with dementia (210). More recently, gait study has incorporated more accurate and sophisticated measurement systems, showing that gait assessment is a more complex multidimensional construct than the gait speed. For instance, Ijmker and Lamoth (211) found that during walking (single task) and walking while performing a letter fluency (dual task) tasks, patients with FTD presented a significantly longer stride time, lower gait speed, and higher stride variability than healthy older adults. In another study, Rucco et al. (212) found that patients with bvFTD performing single and dual tasks (walking while serially subtracting 7s starting from 100) present a significant difference in gait velocity (speed, stride length, cadence) and instability (stance time, swing time) compared to the healthy group.

Despite the scarcity of research regarding gait assessment in FTD (213), it has shown to be critical when differentiating between neurodegenerative diseases. For instance, the study developed by Allali et al. (214) found that patients with bvFTD showed an increase in stride time coefficient variation during a single (walking) and dual tasks (walking and counting backward by one) in comparison to the AD group. A longitudinal study developed by de Cock et al. (215) found multiple significant associations between different components in gait assessment and the future dementia type (AD, FTD, VD, and LBD).

Despite the increasing evidence demonstrating the potential of gait assessment for the diagnostic discrimination between FTD and other dementias, there is no study of these features in LAC.

#### Behavior and Neuropsychiatric Symptoms

The core of bvFTD are behavioral features, as stated in the Current Consortium Criteria for bvFTD (67). These symptoms must present within the first 2–3 years from the onset of disease. Onset is insidious and these features are usually reported by family members or caregivers, as the patients often lack insight. Disinhibition is one of the prominent symptoms and is evident in 76% of the cases. It is manifested through impulsivity, inappropriate social behavior, and lack of decorum. Apathy, the other predominant feature, reaches 84% of the cases, presenting inertia and a lack of motivation. Loss of empathy and/or sympathy and stereotyped behaviors are frequent manifestations reaching up to 70% of patients with bvFTD, while almost 60% of cases present eating disturbances (67, 216). Psychotic symptoms, such as delusions and hallucinations, have been described as less commonly (217). One study reported that 14% of patients with FTD presented delusions, mostly of a paranoid or somatic type (218).

Several assessments, mostly caregiver-based questionnaires, have been used to evaluate neuropsychiatric and behavioral symptoms in FTD. One of them is the Frontal Behavioral Inventory (FBI), which can help to distinguish FTD from other types of dementia but cannot differentiate between bvFTD and psychiatric conditions (219). Nevertheless, sub items such as indifference/emotional flatness, inappropriateness, aphasia, verbal apraxia, alien hand, and apraxia are more suggestive of bvFTD (220). The Frontal Systems Behavior Scale (FrSBe) is another test designed to evaluate apathy, executive dysfunction, and disinhibition (221). The Cambridge Behavioral Inventory Revised (CBI-R) is a questionnaire evaluating a wide range of neuropsychiatric features and everyday functionality. This test was able to discriminate the behavioral profiles of the various neurodegenerative diseases, including AD, Parkinson's Disease (PD), and bvFTD (222, 223). The Neuropsychiatric Inventory Questionnaire (NPI-Q) (224), a short version of the Neuropsychiatric Inventory (NPI) (54), is a tool used to evaluate neuropsychiatric symptoms and response to treatment in patients with dementia, and it has also been used for bvFTD. A behavioral inventory based on the current International Consensus Criteria, DAPHNE (225), allows differentiating the bvFTD from the bipolar disorder. Ducharme et al. (226) developed a 17-item tool, the FTD vs Primary Psychiatric Disorder Checklist, which may be useful in clinical settings and showed good diagnostic accuracy.

There are several scales for more specific symptoms, such as: (a) Apathy may be assessed by the Apathy Evaluation Scale (AES) (227) or with the Starkstein Apathy scale (SAS) (228); (b) The Stereotypy Rating Inventory (SRI) (229), which recognizes stereotypies as more frequent features in bvFTD than in other conditions; (c) Lack of empathy can be measured by the Interpersonal Reactivity Index (IRI) (230); and (d) The Appetite and Eating Habits Questionnaire APEHQ used to assess dietary disturbances (231).

Several studies in LAC have investigated neuropsychiatric symptoms in FTD. In Brazil (55), the NPI was used to verify accuracy in the differential diagnosis between FTD and AD. The results showed that all patients with FTD and only half of those with AD presented neuropsychiatric symptoms (55). Similarly, another Brazilian study (232) demonstrated the usefulness of the FBI for the differential diagnosis between FTD and AD. In Colombia, the Columbia University Psychopathological Scale for Alzheimer's Disease (CUSPAD) and the NPI were used to assess how neuropsychiatric symptoms could influence cognitive and functional impairment in patients with FTD and AD (56). Another study that assessed apathy using the Starkstein Apathy Scale (SAS) showed that patients with bvFTD had higher scores than healthy controls. In addition, the severity of apathy was associated with a decreased gray matter volume in the midline prefrontal regions (233). A case study of FTD with late-onset compulsions and cinephilia was described by Slachevsky et al. (234). Pathological gambling was also reported in a case with bvFTD (235).

#### Functional Assessment

Impaired ability to carry out ADLs, resulting in a loss of independence, is central to the diagnosis of dementia and establishes the boundary between dementia and pre-dementia (67, 236). Impairment in functional capacity is a common outcome of all dementia syndromes, and their assessment is critical for diagnosing and monitoring disease progression (237). The assessment of functional capacity has focused on the development of objective and sensitive tools (19), which are based on indirect (i.e., informant-based questionnaires) and direct (i.e., performance-based tests) measures (238). These tools assess BADLs, which represent the most basic level of functioning and are necessary for survival, and IADLs, which require more complex skills and enable independent living in the community (19). Recently, Advanced Activities of Daily Living (AADLs) have been incorporated, which are the activities necessary for complex interpersonal and social functioning (239, 240).

This is important considering that the functional decline is present in all types of dementia and that the same functional assessment tools are used for different types of dementia. Research on functional decline assessment in FTD has focused on establishing if there is a specific pattern of functional decline, its progression, associated factors, and its neural basis. Indeed, the rate of functional impairment is marked more significantly in FTD than in AD (17, 237). In this line, one of the research lines has established ADL assessment measures to differentiate between different types of dementia.

In LAC, the study of functionality in FTD is limited. In Argentina (19), the Activities of Daily Living Questionnaire (ADLQ) (241) is available to assess functional impairment in different types of dementia (AD, FTD, and other subtypes). In Chile, the Technology-ADLQ (T-ADLQ) was developed, expanding the ADLQ with an additional subscale to evaluate the use of technology in patients with dementia (AD, FTD, DV, and LBD) (242, 243).

In Brazil, several studies have evaluated the usefulness of different tests: Bahia et al. (232) applied the Disability Assessment for Dementia (DAD) questionnaire (244) for estimating the functional capacity of patients with FTD (bvFTD, SD, and nfv-PPA) and AD, showing promising results. The Direct Assessment of Functional Performance (DAFS) (245) was administered for the study of patients with FTD (238) (unlike the DAD, this is a performance-based test). In addition, Carvalho et al. (246) used the Functional Assessment of Adult Communicative Skills (Asha-Facs) (247) in patients with FTD and AD. The results showed similar performances in both groups of patients (246). Finally, in Chile, the T-ADLQ showed promising results for evaluating functional impairment in FTD (243).

Importantly, all these tools showed good psychometric properties in the applied populations, making them valuable instruments for assessing the functional capacity of patients with FTD in LAC (19, 246). These instruments are sensitive in identifying impaired functional ability and differentiate patients with dementia from control subjects. Although some tools failed to significantly distinguish between FTD and AD, patients with FTD presented a worse performance in some indices of these scales (238, 246).

Two works explored the association of functional impairment with cognitive and behavioral symptoms in bvFTD. A multicentric study in Brazil, Australia, England, and India (20) showed an association between impairment in a global functional capacity and IADLs, evaluated through the DAD, with global cognitive impairment and apathy (20). More recently, a study explored factors associated with domains of functional impairment as assessed with the T-ADLQ [i.e., BADLs, IADLs, and AADLs (243)]. Interestingly, factors associated with the loss of functionality differ according to the functional domain, i.e., impairments in IADLs were associated with apathy and disinhibition, in IADLs with apathy, deficits in executive function, lack of emotion recognition, and in IADLs with apathy. This study suggested that the factors associated with loss of functionality differ according to the functional domain in patients with bvFTD in its early stage, along with a prominent and transverse effect of apathy in the loss of functionality throughout all the ADL domains, and the association of social cognition with functional impairment (243).

#### Global Rating Scale

Global assessment scales allow clinical characterization and longitudinal assessment of patients with neurodegenerative diseases (248). In addition, these scales allow proper clinical management and personalized care of patients with dementia, monitoring the progression of the disease and the effects of treatments that could modify the course of the illness (249).

The main instrument used for the global classification of dementia is the Clinical Dementia Rating (CDR) (250), which provides information on cognitive and functional aspects of the disease (251). The CDR is a semi-structured interview administered to the patient and to the primary caregiver, which provides information on six specific domains (memory, orientation, judgment and problem solving, community affairs, home, hobbies, and self-care). Each domain and the scale as a whole reports values ranging from low to high severity: 0 (no impairment), 0.5 (very mild), 1 (mild), 2 (moderate), and 3 (severe) (252). However, the CDR was developed based primarily on AD symptoms, making it a less sensitive scale for other types of dementia, such as FTD (30, 249, 253).

To address the low sensitivity of the CDR, Knopman et al. (254) proposed a new version, the Clinical Dementia Rating Scale for Frontotemporal Lobar Degeneration (CDR-FTLD). This scale incorporated language and behavioral domains (249, 252), providing specific information on FTD and its variants (252). On the other hand, Mioshi et al. (30) proposed a specific scale for FTD, the Frontotemporal Dementia Rating Scale (FTD-FRS). The FTD-FRS was designed based on the DAD and the Cambridge Behavioral Inventory (CBI). This scale allows staging the severity of FTD in its different variants, such as bvFTD and PPA (30, 252, 253).

In LAC, the CDR-FTLD was adapted and validated in Argentina (251) and Brazil (249). Lima-Silva et al. (252, 253) translated, adapted, and validated the FTD-FRS into Portuguese and administered it together with the FTD-FRS and the CDR to patients with FTD (bvFTD, PPA), AD, and healthy controls. In these studies, the CDR was observed to underestimate FTD severity, as it classified patients with mild severity (CDR = 1), unlike the FTD-FRS, which indicated moderate levels of severity (253). The same Lima-Silva group evaluated the ability of the FTD-FRS in comparison with the CDR-FTLD and CDR to detect the functional and behavioral changes in patients with bvFTD, PPA, and AD after 12 months of follow-up (249). All three scales detected an increase in symptom severity after the initial assessment. However, the FTD-FRS and CDR-FTDL were more sensitive in establishing the severity level in bvFTD and PPA (249).

In conclusion, global staging scales used to assess FTD in LAC can determine the stage and progression of the disease by identifying changes in behavior and language that the CDR does not consider (253). Therefore, these instruments are appropriate for clinical use in addition to being well-tolerated by patients and their caregivers (253). Finally, global rating scales show excellent psychometric and diagnostic properties for assessing FTD and its spectrum in LAC.

### Proposition for the Multidimensional Clinical Evaluation of FDT and Its Spectrum in LAC

Considering the evidence on the adaptation, validity, diagnostic utility, and standardization in LAC of the reviewed instruments, we propose a multidimensional clinical assessment and the identification of gaps that represent essential barriers for a comprehensive evaluation of FTD. Importantly, cognitive assessment could be limited to cognitive screening in patients with mild symptoms or with a well-established diagnosis in whom further assessment will not contribute to the diagnosis. Otherwise, we recommend a multidimensional evaluation organized in three steps: (1) Tests to be administered to all patients regardless of variant; (2) Specific tests for specific variants, i.e., language or behavior; and (3) Additional tests for the assessment of specific symptoms.

In Table 1, we propose tests for the first level of the multidimensional clinical assessment. The first step, the tests to be administered to all patients, allows assessing the fundamental dimensions for a proper diagnosis of FTD, i.e., cognition, functionality, neuropsychiatry, and motor symptoms. Significantly, clinical symptoms reported by people with dementia and/or a reliable proxy do not necessarily predict the pattern of cognitive impairment or whether they are preserved (255). Therefore, assessing the main cognitive domains in all patients with suspected FTD is necessary to establish the pattern of cognitive impairment correctly.

**Table 1:** Tests to be administered to all patients regardless of variant.

**Tests to be administered to all patients regardless of variant**			
	**Recommendations: test or assessments**	**Recommendations: research**	Recommendations: clinical^*^
Global cognitive screening	ACE-III	Need for adaptation, validation, and standardization in several LAC countries Need for validation in low educational levels and in indigeneous populations ACE III may be used to compare LAC populations	ACE III should be complemented with an Executive Screening Not appropriate for evaluating the illiterate population
Frontal screening	IFS	Need for adaptation, validation, and standardization in several LAC countries Need for validation in low educational levels and in indigeneous populations Cultural adaptation of the proverbs section	IFS should be complemented with a Global Cognitive Screening
Episodic memory	RAVLT FCSRT pictorial and verbal	Need for adaptation, validation, and standardization in several LAC countries Need for validation in low educational levels and in indigeneous populations Need for studies to assess sensitivity in FTD	These instruments allow differentiating the processes of encoding, storing, and retrieving learned information. This differentiation is necessary to show the FTD performance profiles and their spectrum
Language: fluency	Phonological Fluency Categorical Fluency	Need for adaptation, validation, and standardization in several LAC countries Need for validation in low educational levels and in indigeneous populations	In case of evaluation time limit, ACE-III fluency task can be used
Denomination	BDAE (30 items)	Need for adaptation, validation, and standardization in several LAC countries Need for validation in low educational level	In case of evaluation time limit, ACE III denomination stimuli can be used
Praxis	No specific task can be recommended at this time	Need for adaptation, validation, and standardization for this specific cognitive domain in LAC countries	Praxis requires evaluation. Although no evaluation instrument is recommended, evaluating gestures with and without meanings is suggested to obtain clinical information
Semantic memory	ACE-III: 4 semantic memory stimuli as an index	Need for adaptation, validation, and standardization for this specific cognitive domain in LAC countries Need for a reliable semantic memory index Need for validation in low educational levels and in indigeneous populations	If the ACE-III index of semantic memory is altered, explore semantic memory with more specific tests We recommend caution when interpreting the result of these tests due to the importance of socio-cultural factors in semantic memory
Visuoconstructive abilities	ROCF: Copy	Need for adaptation, validation, and standardization in several LAC countries Need for validation in low educational levels and in indigeneous populations	Evaluate final score and strategies used to estimate planification figure construction Simple figures of ACE-III can be used to evaluate this cognitive domain
Visual memory	ROCF: memory	Need for adaptation, validation, and standardization in several LAC countries Need for validation in low educational levels and in indigeneous populations	ROCF copy score is necessary for the interpretation of the scores
Executive function	Phonological Fluency Categorical Fluency M-WCST Hayling Test TMT A and TMT B TMT Color	Need for adaptation, validation, and standardization in several LAC countries Need for validation in low educational levels and in indigeneous populations	Apply Verbal Control Inhibitory subtest of IFS in case there is no access to Hayling Test IFS subtest can be used to evaluate Working Memory TMT-A can be used to assess information processing speed Use Color version of TMT for low educational levels
Social cognition	Mini-SEA Subtest: Faux Pass Subtest: Face Recognition	Need for adaptation, validation, and standardization in several LAC countries Need for validation in low educational levels and in indigeneous populations Separate research about the diagnostic value of Mini-SEA and its subtests Study on the clinical utility of tests with high ecological validity to predict social behavioral disorders in research Need for clinically validated instruments to assess other areas of social cognition such as empathy and moral emotion	In Faux Pass: use clear and standardized instructions for this task, specifically explain that the questions are about social norms and not about personal opinions. Also, the control questions evaluate comprehension for the total score result MiniSea is not suitable for the illiterate and low-educated population
Gait assessment	Single task Dual task (cognitive task while person is walking)	Need for adaptation, validation, and standardization for this specific domain in LAC countries Need for quantitative gait measurement studies for FTD and its spectrum	Gait Assessment should be complemented with a Cognitive Screening
Neuropsychiatric assessment	FBI FrSBe NPI-Q	Need for adaptation, validation, and standardization in several LAC countries Need for validation in low educational levels and in indigeneous populations Need for cross-cultural validation of the diagnostic utility of FBI and FrSBe.	FBI is a good tool to structure the clinical interview The long time required to administer FrSBe limits its incorporation into clinical practice
Functional assessment	T-ADLQ DAD	Need for adaptation, validation, and standardization in several LAC countries	T-ADLQ is a good tool to structure clinical interview
Global rating scale	CDR-FTLD FTD-FRS CDR	Need for adaptation, validation, and standardization in several LAC countries	If the CDR (focused on AD assessment) is applied, it is necessary to add the CRD-FTLD language and behavioral task

* *Clinical recommendations are based on the knowledge acquired during daily practice over several years by the experts who constructed this recommendation table*.

*ACE-III, Addenbrooke's Cognitive Examination—Third version; IFS, INECO Frontal Screening; RAVLT, Rey Auditory-Verbal Learning Test; FCSRT, Free and Cued Selective Reminding Test; BDAE, Boston Diagnostic Aphasia Examination; ROCF, Rey-Osterrieth Complex Figure; M-WCST, Modified Wisconsin Card Sorting Test; TMT, Trail Making Test; Mini-SEA, Social Cognition and Emotional Assessment; FBI, Frontal Behavioral Inventory; FrSBe, Systems Behavior Scale; NPI-Q, The Neuropsychiatric Inventory Questionnaire; T-ADLQ, The Technology - Activities of Daily Living Questionnaire; DAD, Disability Assessment for Dementia; CDR-FTLD, Dementia Rating Scale for Frontotemporal Lobar Degeneration; FTD-FRS, Frontotemporal Dementia Rating Scale; CDR, Clinical Dementia Rating; LAC, Latin America and the Caribbean; FTD, frontotemporal dementia; AD, Alzheimer's Disease*.

In Table 2, corresponding to the second level, some of the recommended instruments have been widely used for clinical assessment and investigation of FTD in LAC. However, we must mention that the results of our review suggest that in most LAC countries, there is no information on the adaptation, validation, and standardization of these instruments. Additionally, the diagnostic utility of these tools has been studied mainly for AD but not for other subtypes of dementia. This second step involves specific testing for the different variants of FTD, i.e., behavioral or language variants. Finally, the third step should include evaluating some patients with more atypical or complex presentations who will benefit from additional testing. However, it is challenging to recommend further testing for these atypical presentations. Therefore, more research is needed.

**Table 2:** Specific tests for specific variants of FTD.

**Specific tests for specific variants of FTD**			
	**Recommendations: test or assessments**	**Recommendations: research**	Recommendations: clinical^*^
Language variants	BDAE Sydbat PPT RPT	Need for adaptation, validation, and standardization in several LAC countries Need for validation in low educational levels and in indigeneous populations Development of instruments for language variants of FTD suitable for LAC	RPT may differentiate between nfv-PPA and SD
Behavioral variant	SEA Mind in the eyes	Need for adaptation, validation, and standardization in several LAC countries Need for validation in low educational levels and in indigeneous populations	In case of diagnostic doubt, a complementary evaluation is suggested To complement evaluation of Social Cognition apply SEA with Mind in the Eye To complement evaluation of executive functions apply the Hotel Test

* *Clinical recommendations are based on the knowledge acquired during daily practice over several years by the experts who constructed this recommendation table*.

*BDAE, Boston Diagnostic Aphasia Examination; Sydbat, Sydney Language Battery; PPT, Pyramids and Palm Trees Test; RPT, Repeat and Point Test; SEA, Social cognition and Emotional Assessment; LAC, Latin America and the Caribbean; FTD, Frontotemporal dementia; nfv-PPA, non-fluent or agrammatical aphasia; SD, semantic dementia*.

## Discussion

Notably, our review suggests an important variety of practices in the assessment of FTD in LAC. The recommendation of a comprehensive multidimensional assessment of FTD is limited due to the existence of the main knowledge gaps that could be divided into three main areas. First, there is a lack of validated cognitive, functional, behavioral, and motor instruments for diagnosing FTD. Second, there are almost no tools to evaluate the illiterate and indigenous population. Third, there are no guidelines to orient clinicians on which patients would benefit from a multidimensional assessment. Finally, we will propose how to address the future challenges.

### Domains Without Adequate Assessment Tools

To the best of our knowledge, there are no properly adapted and validated tests for assessing semantic memory and social cognition in LAC. The available tools raise doubts about their validity and diagnostic utility. Currently, social cognition is primarily assessed with the Mini-SEA. Although there are promising results on the diagnostic utility of the Mini-SEA for the differential diagnosis of bvFTD of PD and AD (61, 198), social cognition assessment still faces essential limitations.

The investigation of behavioral and neuropsychiatric symptoms is of utmost importance for the correct diagnosis of FTD. While behavioral and psychiatric scales are of value for screening and measuring these symptoms, the cultural context should also be considered in the neuropsychiatric assessment. Indeed, the examiner may perceive some characteristics of interpersonal interaction as “normal” or “abnormal” according to cultural, personal, and social factors. For instance, interpersonal distance and voice volume are features that vary across cultures and may be described as “normal” or “disinhibited” according to the socio-cultural factors. Therefore, it is not enough to have “adapted and validated” tools to measure neuropsychiatric symptoms, but also ways to correctly interpret individual signs in interpersonal interactions in the perspective of a correct clinical diagnosis.

We think it is important to emphasize that gait dysfunction and, more generally, motor dysfunction have a large amount of overlap in genetics and molecular biology with cognitive disorders (256). Nevertheless, they are not part of the routine assessment of patients with dementia (257). This situation must be improved given that, for example, Parkinson's disease dementia (PPD), PSP, CBS, and Huntington's disease (HD), among others, present motor impairments as their main clinical features. Indeed, the apraxia profile or the applause sign could contribute to the differential diagnosis of diseases included in the FTD spectrum (258). Therefore, this manuscript proposes a gait assessment based on quantitative assessment systems (e.g., 3D motion capture, 2D kinematics, and spatiotemporal gait analysis system). However, the main difficulty in incorporating these systems is that they are expensive, making them difficult to access in the hospitals and clinics in LAC. A viable and much more inexpensive alternative is the wearable devices for gait analysis. Recent studies have found that wearable devices can differentiate gait alteration in dementia disease subtypes (259, 260). Nevertheless, we must remain cautious regarding this wearable technology because they have shown limitations in quantifying gait (e.g., the diversity in the sensor placements and the abundance of inertial algorithms) (203).

Unfortunately, as far as we know, there is no validated brief tool for motor assessment in FTD and its spectrum (256, 261). Concerning gait, we still require systematic studies to understand its contributions in FTD diagnosis. Ideally, a motor assessment tool in patients with dementia should include assessment of the gait pattern, parkinsonian gait, cerebellar gait, and higher-order symptoms such as praxis and motor sequencing (261). Such a tool would most likely benefit from the incorporation of wearable devices that could allow a more objective measurement of motor impairment.

Finally, it is important to highlight that the tools reviewed here have been mostly validated in studies with clinical-based FTD diagnoses without a pathological diagnosis confirmation. Considering a huge amount of evidence suggesting that FTD-related clinical syndromes are associated with heterogeneous pathology (262), it is important to emphasize that recommended tests allow prediction of a clinical syndrome, but not of a given specific pathological diagnosis (263). Either way, predicting neuropathology is beyond the scope of neuropsychology, and an etiopathogenic diagnosis of FTD requires a multilevel assessment including clinical, neuroimaging, and molecular biomarkers (264).

In sum, the translation and the validation of neuropsychological tests and their cultural adaptation are warranted to improve cognitive, functional, behavioral, and motor assessment of patients with FTD in LAC.

### Patient Selection for Assessment

As suggested in international consensus studies, it is advisable to follow a multi-step approach to define the proper flow for each patient (261, 265). The first step should be applying a brief global screening instrument to all subjects with suspected cognitive impairment. Global tools, such as the ACE-III and the IFS, are recommended in the Spanish and Portuguese-speaking population (see Table 1). If these instruments and the clinical assessment suggest cognitive impairment and a diagnostic doubt persists, a multidimensional assessment should be performed (266).

Nevertheless, there are no clear guidelines on which patients would benefit from a multidimensional assessment. Considering the barriers to access specialist bvFTD evaluation centers (42, 267), the diagnosis process of bvFTD could be improved with the availability of evidence-based guidelines to help identify patients that could benefit from a multidimensional assessment.

### Populations for Which We Need Better Assessment Tools

Most of the instruments that have been validated in LAC are specifically for a literate population with, in general, a minimum of 4 years of education, which presents a significant drawback for the assessment of the illiterate and low-educated population (268). The absence of validated tests for the low-educated population is a significant limitation in assessment since years of education and age are two of the main variables affecting performance in cognitive assessments (261). Educational level affects instruments with low specificity given the difficulty in classifying subjects who possess diminished academic levels and how these patients obtain low scores despite being healthy. This situation also occurs with low-sensitivity instruments. Classifying subjects with a high academic level and high scores can be difficult despite presenting cognitive impairment (261).

Indeed, almost 4% of the illiterate population or with very low education levels of the world is found in LAC (269). Functional illiteracy is significant in LAC (270). In addition, about 10–17% of the LAC population is indigenous, with an estimated 400 indigenous languages spoken, along with Spanish and Portuguese (271). Finally, there is an increased percentage of non-Spanish or Portuguese-speaking migration (268). For example, in Chile, a large population of Creole-speaking Haitian citizens has recently arrived in the country, which generates a challenge and a limitation regarding the tools currently used in Chile. Economic factors should also be considered when proposing tests for these populations, as their financial vulnerability hampers access to expensive assessments.

### Consortium for Multidimensional Assessment of FTD

In LAC, research and clinical evaluation of FTD and its spectrum have been conducted by a small group of professionals who share common needs and interests (61, 272). Nevertheless, transfer from research to clinical practice is restricted and significant knowledge gaps limit the implementation of multidimensional assessments. Following multicentric and multi-country initiatives in Europe and North America to improve assessment of neurodegenerative diseases, we propose the creation of a LAC consortium as the best strategy to address current challenges in the multidimensional clinical assessment of FTD. In fact, we are not aware of any organized working group to transfer research to a clinical practice. Regarding the clinical practice, there is currently an enormous heterogeneity of tools used in different countries, a lack of standardization of administration and scoring methods, and scarce information on the psychometric properties and diagnostic utility of some instruments. Moreover, the number of reliable instruments to assess the different dementias is limited, and there is no consensual evaluation protocol (261, 273). This problem directly affects the study of FTD and its spectrum, hindering the advancement of clinical and research practice in this type of dementia and not allowing the comparison and sharing of results from different studies conducted in LAC.

As suggested by international initiatives on the dementia assessment (261, 274), the formation of a consortium to share the works of professionals within LAC is probably the best strategy to establish a consensual multidimensional evaluation of FTD and its spectrum, and to overcome the shortcomings and the regional needs. A key point, as widely discussed and demonstrated in international consensus studies for dementia evaluation (261, 274), is the need for evaluation protocols that are consensual and homogenized by different countries and their local study centers. In addition, these evaluation protocols must have a standardized administration and a scoring procedure (274). In this line, the necessity of a standardized evaluation responds to the different backgrounds of the professionals who apply the evaluation instruments, including neuropsychologists, speech therapists, nurses, occupational therapists, and physicians (274). The contexts where the evaluation instruments are applied are also varied, such as primary care facilities, memory clinics, specialized centers, or in a research context (261).

A homogeneous evaluation practice based on a professional consensus for the assessment of cognitive, functional, behavioral, and motor abilities of patients with dementia (261) could guide the framework for different professionals, generating knowledge and shared data repositories of FTD studies and its spectrum in LAC. This effort could be critical for advancing studies on the adaptation, validation, and standardization of assessment tools (which are critical for the correct interpretation of study results) and possible educational processes and training for LAC professionals. Additionally, a homogenous evaluation practice could enable providing guidelines for implementing a multiple step approach in the evaluation. This is particularly relevant in LAC considering the lack of knowledge on FTD and its spectrum in health professionals (40, 61). In this effort, integrating clinical practice and research is relevant for generating new knowledge to evaluate the clinical utility of a multidimensional assessment, identifying patients that could benefit from this assessment, and elaborating the evidence-based guidelines to define the correct flow for each patient.

#### Steps for the Development of a Consensus

International evidence regarding consortiums highlights the main steps to succeed in the establishment of a definitive consensus. European experience suggests that the first step is the creation of a working group or a consortium that brings together different researchers and clinicians (neurologists, neuropsychologists, occupational therapists, speech therapists, and among others) from different countries (261). Each country should have one or two representatives from their main centers of dementia care or research who have specific skills in the diagnosis and evaluation of FTD and its spectrum. These representatives should be available to participate in periodic online working sessions. A general organization of the work plan should be established as follows: (i) Review the totality of assessment tools available in the different LAC centers, (ii) Define a global screening assessment for patients with FTD, and (iii) Establish a detailed assessment of the different variants of FTD covering cognitive, functional, behavioral, and motor dimensions.

Researchers or clinicians from different LAC countries, separated in groups, will seek which assessment tools are currently available to study cognitive, behavioral, functional, and motor dimensions. They will search for the psychometric properties (validity and reliability) and diagnostic utility (sensitivity and specificity) of the tools, their main issues, and propose solutions to solve the respective issues. The information obtained from the different working groups will allow for establishing a definitive consensus and develop a standardized evaluation protocol, which will indicate the instruments to be used in each dimension. This standardized protocol will allow the different centers studying patients with FTD in LAC to use a similar method of data collection. It will also allow the development of training and education processes for professionals through websites and free access to manuals and instruments that will have to be adapted and validated in different cultures.

A common methodology should be proposed regarding the adaptation, validation, and standardization of the evaluation instruments. Establishing a strategy is necessary for carrying out these studies among the different LAC countries, allowing the development of multicenter FTD data repositories. Finally, the support and financing of local and international initiatives should be sought out, along with the support and advice of different consensuses carried out in different parts of the world. This will help our local initiative to be carried out successfully. As seen in the international experience, the way to carry out these initiatives starts from formal entities that have sufficient funding to execute consensus regarding the evaluation of patients with dementia (261, 265). This same idea could be replicated in LAC, seeking entities or creating a consortium that can lead this process and establish a multidimensional clinical assessment in FTD and its spectrum. Initiatives, such as the Multi-Partner Consortium to Expand Dementia Research in Latin America (ReDLat), the Latin America and the Caribbean Consortium on Dementia (LAC-CD), or the United Kingdom–Brazil Dementia Workshop, could constitute the first step in this effort.

## Conclusion

Our paper is the first joint initiative to establish a multidimensional clinical assessment for FTD and its spectrum in LAC. Our proposal provides valuable input to a future consortium and to the different LAC countries to adopt a uniform assessment method that considers the different local realities of each country.

**Figure 1:**
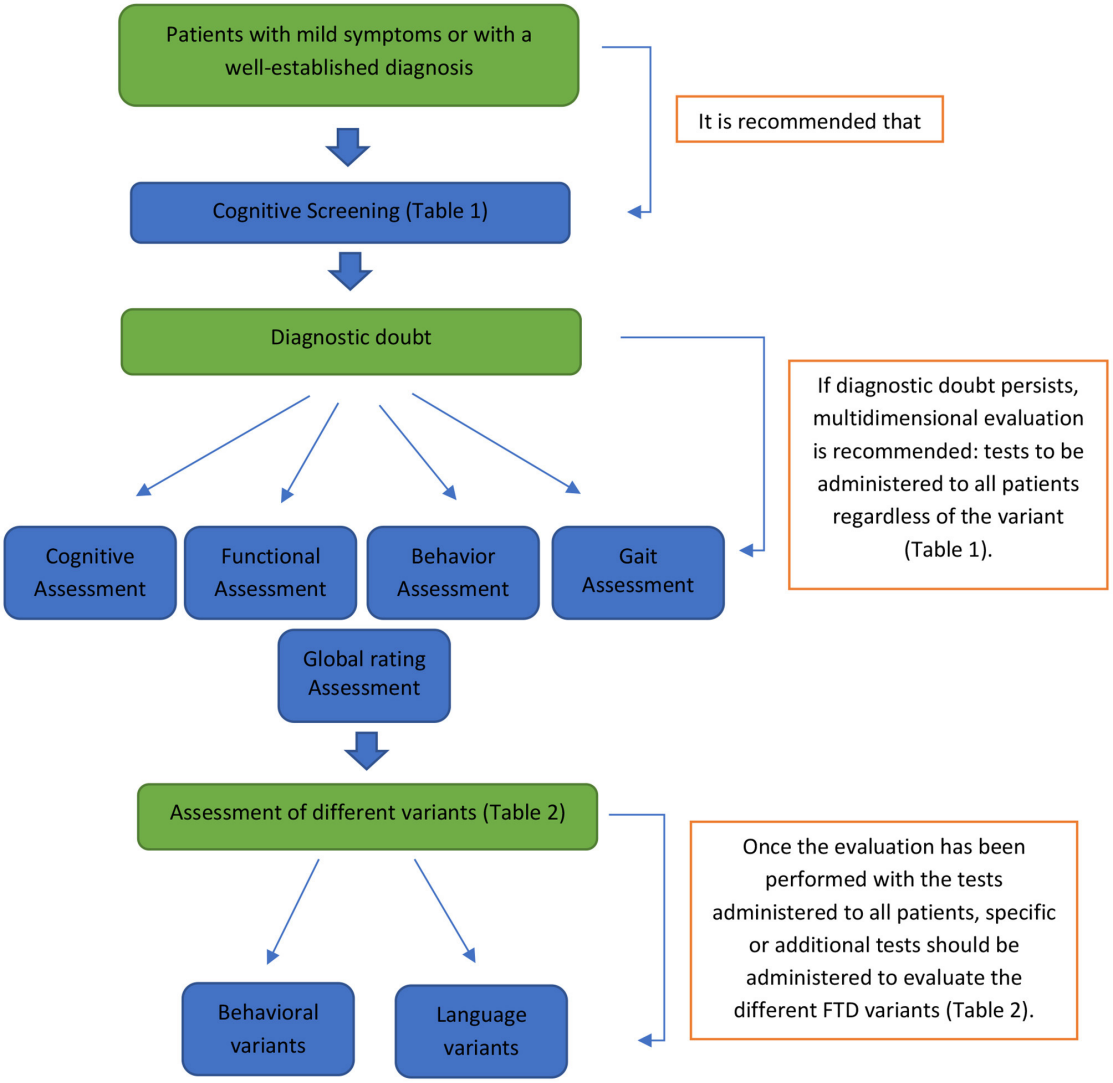
Preliminary standard assessment protocol. This protocol shows the different phases and evaluations to which each patient should be submitted according to the clinical characteristics presented. Suppose we are in the presence of a patient with mild symptoms or with a well-established diagnosis. In that case, it is advisable to evaluate with screening tools (see tools in Table 1). If there is diagnostic doubt, the patient should undergo a multidimensional evaluation (cognitive, functional, behavioral, and motor; see tools in Table 1). After this last step, the administration of additional assessment instruments associated with the specific variant of FTD studied is suggested (see tools in Table 2).

The multidimensional assessment proposal, which arises from the published evidence and the recent experiences in FTD studies in LAC, allows the establishment of a preliminary standard assessment protocol for this region (see Figure 1). This protocol aims to assess the primary cognitive, functional, behavioral, and motor domains altered in FTD and its spectrum, which can be used to study patients with suspected or established diagnoses. The proposed protocol is broad enough to contribute to the clinical differentiation between FTD and other types of dementia. It could also help differentiate FTD from psychiatric pathologies.

Although this work does not provide information on the normative and psychometric data, or diagnostic utility of all the recommended instruments, it is a first compilation of the minimal and necessary tools for the assessment of FTD. Importantly, valid and reliable tools are recommended in the assessment and follow-up of patients with dementia according to the international evidence.

Patients with FTD and its spectrum face difficulties in access to diagnosis, thereby increasing the burden on patients and their caregivers (267). Therefore, promoting a consensual and multidimensional assessment of FTD and its spectrum through an LAC consortium with validated and reliable tools for the main clinical dimension of FTD, i.e., cognition, functional, behavioral, and motor, could contribute toward addressing diagnosis barriers. The implementation of a multidimensional assessment requires the joint effort of an interdisciplinary team involving physicians, neuropsychologists, occupational therapists, speech therapists, kinesiologists, among others, working to foster both research and sharing of clinical practices. A consortium that brings together an interdisciplinary group represents the best strategy to create the knowledge necessary to facilitate access to diagnosis for patients with FTD in LAC, and to become a more equitable community with better capabilities when facing FTD and its spectrum. Finally, a similar effort is much more needed for dementia in general and its different types, for which we also lack a common approach in LAC.

## Author Contributions

FH and AS designed the proposal. FH, VC, SB, LS, PL, DM-P, LO, TT, and AS wrote the drafts and discussed contributions from all co-authors. All authors participated in discussing the contents of the paper, contributed to editing, and approved the final version of the article.

## Funding

AS, PL, and DM-P were supported by grants from ANID/FONDAP/15150012. FH, VC, DM-P, LO, and AS were supported by ANID/FONDEF/ID 18I10113. FH, VC, and AS were supported by ANID/Fondecyt/1191726, 1210176, and 1210195 and MULTI-PARTNER CONSORTIUM TO EXPAND DEMENTIA RESEARCH IN LATIN AMERICA [ReDLat, supported by National Institutes of Health, National Institutes of Aging (R01 AG057234), Alzheimer's Association (SG-20-725707), Tau Consortium, and Global Brain Health Institute], and Alzheimer's Association GBHI ALZ UK-20-639295. FH was supported by grants from ANID-Subdirección de Capital Humano/Doctorado Nacional/2021- 21211349. LS was supported by the Brazilian National Council for Scientific and Technological Development (CNPq—*bolsa de produtividade em pesquisa*).

## Author Disclaimer

The contents of this publication are solely the responsibility of the authors and do not represent the official views of these institutions.

## Conflict of Interest

The authors declare that the research was conducted in the absence of any commercial or financial relationships that could be construed as a potential conflict of interest.

## Publisher's Note

All claims expressed in this article are solely those of the authors and do not necessarily represent those of their affiliated organizations, or those of the publisher, the editors and the reviewers. Any product that may be evaluated in this article, or claim that may be made by its manufacturer, is not guaranteed or endorsed by the publisher.

## Abbreviations

AADL: Advanced Activities of Daily Living
ACE-III: Addenbrooke's Cognitive Examination - Third version
ACE-R: Addenbrooke's Cognitive Examination - Revised
AD: Alzheimer's disease
ADLQ: Activities of Daily Living Questionnaire
ADL: Activities of Daily Living
AES: Apathy Evaluation Scale
ALS: Amyotrophic Lateral Sclerosis
APEHQ: Appetite and Eating Habits Questionnaire
Asha-Facs: Functional Assessment of Adult Communication Skills
BADL: Basic Activities of Daily Living
BADS: Behavioral Assessment Dysexecutive Syndrome
BCS: rief Cognitive Screening
BDAE: Boston Diagnostic Aphasia Examination
BEM 144: Signoret battery for mnesic efficiency
bvFTD: behavioral variant of FTD
BVRT: Benton Visual Retention Test
CAMPROMPT: cambridge Behavioral Prospective Memory Test
CBI: Cambridge Behavioral Inventory
CBI-R: Cambridge Behavioral Inventory Revised
CBS: Corticobasal syndrome
CDR: Clinical Dementia Rating;
CDR-FTLD: Clinical Dementia Rating Scale for Frontotemporal Lobar Degeneration;
CDT: Clock Drawing Test;
CUSPAD: Columbia University Psychopathology Scale for Alzheimer's Disease
CVLT: California Verbal Learning Test
DAD: Disability Assessment for Dementia
DAFS: Direct Assessment of Functional Performance
FAB: Frontal Assessment Battery
FBI: Frontal Behavioral Inventory
FCSRT: Free and Cued Selective Reminding Test
FrSBe: Frontal Systems Behavior Scale
FTD: Frontotemporal Dementia
FTD-FRS: Frontotemporal Dementia Rating Scale
FTD-MND: FTD with motor neuron disease
HD: Huntington's disease
HVLT-R: Hopkins Verbal Learning Test
IADL: Instrumental Activities of Daily Living
IFS: INECO Frontal Screening
IGT: Iowa Gaming Test
IRI: Interpersonal Reactivity Index
LAC: Latin America and the Caribbean
LBD: Lewy Body Dementia
lv-PPA: Logopenic variant of PPA
MDRS: Mattis Dementia Rating Scale
Mini-SEA: Social cognition and Emotional Assessment - short version
MMSE: Mini-Mental State Examination
MoCA: Montreal Cognitive Assessment
M-WCST: Modified Wisconsin Card Sorting Test
nfv-PPA: Non-fluent or agrammatical variant of PPA
NPI: Neuropsychiatric Inventory
NPI-Q: Neuropsychiatric Inventory Questionnaire
PAL: Paired Associate Learning Test
PD: Parkinson's Disease
PFAQ: Pfeffer Functional Activities Questionnaire
PPA: Primary progressive aphasia
PPD: Parkinson's disease dementia
PPT: Pyramids and Palm Trees Test
PSP: Progressive supranuclear palsy
RAVLT: Rey Auditory-Verbal Learning Test
RBMT: Rivermead Behavioral Memory Test
ROCF: Rey-Osterrieth Complex Figure
RPT: Repeat and Point Test
SAS: Starkstein Apathy Scale
SD: Semantic Dementia
SEA: Social cognition and Emotional Assessment
SRI: Stereotypy Rating Inventory
STMB: Short-Term Memory Binding
SYDBAT: Sydney Language Battery
T-ADLQ: Technology-ADLQ
TMT: Trail Making Test
VD: Vascular Dementia
WAIS: Wechsler Adult Intelligence Scale
WCST: Wisconsin Card Sorting Test
WMS: Wechsler Memory Scale
VOSP: Visual Object and Space Perception Battery.
